# 3,5-Diiodo-L-Thyronine Affects Structural and Metabolic Features of Skeletal Muscle Mitochondria in High-Fat-Diet Fed Rats Producing a Co-adaptation to the Glycolytic Fiber Phenotype

**DOI:** 10.3389/fphys.2018.00194

**Published:** 2018-03-09

**Authors:** Elena Silvestri, Federica Cioffi, Rita De Matteis, Rosalba Senese, Pieter de Lange, Maria Coppola, Anna M. Salzano, Andrea Scaloni, Michele Ceccarelli, Fernando Goglia, Antonia Lanni, Maria Moreno, Assunta Lombardi

**Affiliations:** ^1^Department of Science and Technologies, University of Sannio, Benevento, Italy; ^2^Department of Biomolecular Sciences, Urbino University, Urbino, Italy; ^3^Dipartimento di Scienze e Tecnologie Ambientali, Biologiche e Farmaceutiche, Università degli Studi della Campania, Caserta, Italy; ^4^Proteomics & Mass Spectrometry Laboratory, ISPAAM, National Research Council, Naples, Italy; ^5^Department of Biology, University of Naples Federico II, Naples, Italy

**Keywords:** skeletal muscle, proteomics, mitochondrion, diiodothyronine, reactive oxygen species, inflammation, mitochondrial dynamics, adipose differentiation-related protein

## Abstract

Hyperlipidemic state-associated perturbations in the network of factors controlling mitochondrial functions, i. e., morphogenesis machinery and metabolic sensor proteins, produce metabolic inflexibility, insulin resistance and reduced oxidative capacity in skeletal muscle. Moreover, intramyocellular lipid (IMCL) accumulation leads to tissue damage and inflammation. The administration of the naturally occurring metabolite 3,5-diiodo-L-thyronine (T2) with thyromimetic actions to high fat diet (HFD)-fed rats exerts a systemic hypolipidemic effect, which produces a lack of IMCL accumulation, a shift toward glycolytic fibers and amelioration of insulin sensitivity in gastrocnemius muscle. In this study, an integrated approach combining large-scale expression profile and functional analyses was used to characterize the response of skeletal muscle mitochondria to T2 during a HFD regimen. Long-term T2 administration to HDF rats induced a glycolytic phenotype of gastrocnemius muscle as well as an adaptation of mitochondria to the fiber type, with a decreased representation of enzymes involved in mitochondrial oxidative metabolism. At the same time, T2 stimulated the activity of individual respiratory complex I, IV, and V. Moreover, T2 prevented the HFD-associated increase in the expression of peroxisome proliferative activated receptor γ coactivator-1α and dynamin-1-like protein as well as mitochondrial morphological aberrations, favoring the appearance of tubular and tethered organelles in the intermyofibrillar regions. Remarkably, T2 reverted the HDF-associated expression pattern of proinflammatory factors, such as p65 subunit of NF-kB, and increased the fiber-specific immunoreactivity of adipose differentiation–related protein in lipid droplets. All together, these results further support a role of T2 in counteracting *in vivo* some of the HFD-induced impairment in structural/metabolic features of skeletal muscle by impacting the mitochondrial phenotype.

## Introduction

Skeletal muscle is a key node in the powerful and complex cross-talk between metabolically active tissues in maintaining energy homeostasis. Excess of dietary nutrients, mainly fats and sugars, may lead to an increased flux of energy fuel substrates and to an augmented lipid burden in peripheral tissues. In skeletal muscle, increased fatty acids uptake and corresponding impaired molecular utilization contribute to the accumulation of lipids leading to lipotoxicity and, thus, to insulin resistance (IR) (Falholt et al., 1988; Levin et al., 2001; Adams et al., 2004; Petersen and Shulman, 2006; Sabin et al., 2007; Koves et al., 2008; Kraegen and Cooney, 2008; Zhang et al., 2010). In skeletal muscle, substantial evidences show that mitochondrial dysfunctions, in terms of number and functionality, are jointly liable for IR (Petersen et al., 2004; Lowell and Shulman, 2005). Skeletal muscle fibers, which are broadly classified as slow twitch (type I) and fast twitch (type II) ones, show remarkable diversity in energy metabolism, plasticity and contractile functions; the former being rich in mitochondria and having high oxidative capacity, the latter generating ATP primarily through glycolysis (Bassel-Duby and Olson, 2006; Schiaffino and Reggiani, 2011). A shift from oxidative to glycolytic fibers takes place in skeletal muscle of type 2 diabetic subjects (Patti et al., 2003; Petersen et al., 2004; Ritov et al., 2005). However, whether this slow to fast switch is deleterious or not remains unclear and still under debate (Turner and Heilbronn, 2008; Muoio, 2010; Muoio and Neufer, 2012). The plasticity of skeletal muscle has been associated with mitochondrial functional/morphological modifications, which allow mitochondria themselves to undertake a fiber type-specific specialization. In the highly regulated molecular network controlling the formation of muscle fibers, coordinating mitochondrial biogenesis and affecting metabolic capabilities of pre-existing mitochondria, a central role is played by peroxisome proliferative activated receptor γ coactivator-1α (PGC-1α). This transcriptional coactivator is generally considered to act as a master regulator of the energy management of skeletal muscle fibers (Lin et al., 2002; Lagouge et al., 2006).

Phenotypic adaptations in response to changes in skeletal muscle functional demand are also orchestrated by mitochondrial dynamics (Dahlmans et al., 2016). Indeed, mitochondria constantly fuse and divide through fusion and fission processes, whose frequencies are balanced to maintain the overall morphology and function of the mitochondrial population (Zorzano et al., 2009). Recent work has shown that obesity and excess energy intake shift the balance of mitochondrial dynamics toward fission, further contributing to mitochondrial dysfunction and IR in skeletal muscle (Jheng et al., 2012). Skeletal muscle mitochondrial oxidative capacity as well as insulin sensitivity and lipid oxidation following chronic high-fat or high-energy feeding can also be impaired by inflammation (Morino et al., 2006; Valerio et al., 2006; Bonnard et al., 2008), with IKK/IκB/NF-κB playing a crucial functional role (Yamamoto and Gaynor, 2001; Yuan et al., 2001; Shoelson et al., 2006). Indeed, free fatty acids activate NF-κB and its nuclear translocation after IκBα phosphorylation and degradation, thus compromising insulin sensitivity in skeletal muscle cells (Sinha et al., 2004; Cai et al., 2005; Barma et al., 2009; Håversen et al., 2009). At the same time, the inhibition of IKK/IκB/NF-κB pathway prevents the free fatty acid-induced impairment of insulin signaling (Moller, 2001; Sinha et al., 2004; Cai et al., 2005; Wei et al., 2008; Barma et al., 2009). Intriguingly, recent studies have used different experimental approaches to address the systemic metabolic dysfunction ongoing in skeletal muscle as elicited by augmented levels and activities of glycolytic fibers (Zhang and Ye, 2012; Holloszy, 2013; Meng et al., 2013). Indeed, several evidences obtained in mice and humans support the notion that increasing muscle mass and glycolytic capacity may effectively counteract IR and type 2 diabetes (T2D) (Zhang and Ye, 2012; Holloszy, 2013; Meng et al., 2013).

Recently, we have shown that the administration of the natural metabolite 3,5-diiodo-L-thyronine (T2) to rats receiving a HFD induces an increase in hepatic fatty acid oxidation, prevents body-weight gain, hypercholesterolemia, and hypertriglyceridemia, in the presence of an unaltered hypothalamus-pituitary-thyroid axis, concomitantly preserving muscle glucose uptake and insulin sensitivity (Moreno et al., 2002, 2011; Lanni et al., 2005; Silvestri et al., 2010; de Lange et al., 2011; Goglia, 2015). At the gastrocnemius muscle level and without inducing sarcopenia (i.e., a known marker of thyrotoxicosis), T2 prevents the HFD-induced IMCL accumulation as well as the derangement in insulin signaling, mainly by inducing a structural and biochemical shift toward glycolytic type II myofibers (Moreno et al., 2011). These results advance the case for a promotion of muscle glycolytic capacity having metabolic benefits. In this study, we have assessed whether the above-mentioned effect of T2 at muscle level may also involve a co-adaptation of the mitochondrial phenotype with respect to corresponding protein profile, biogenesis and dynamic markers, production of reactive oxygen species (ROS), and respiratory chain complex activities, that, in turn, may influence tissue inflammation and lipid storage (i.e., IMCL pools).

## Materials and methods

### Materials

3,5-diiodo-L-thyronine (T2) was purchased from Sigma-Aldrich Corp. (St. Louis, MO). All solvents used were of high-performance liquid chromatography-mass spectrometry (LC-MS) grade (Sigma-Aldrich, St. Louis, MO, USA and Carlo Erba, Milan, Italy). Immobilized pH-gradient (IPG) and ampholites were purchased from Bio-Rad Laboratories, Hercules, CA. Acrylamide, other reagents for polyacrylamide gel preparation, BN-PAGE, and histochemical staining of respiratory complex activity, as well as CHAPS, urea, thiourea, dithioerythriol, EDTA, iodoacetamide, brilliant blue G-colloidal concentrate, and tosyl-phenylalanyl chloromethyl ketone (TPCK)-treated porcine trypsin were from Sigma-Aldrich. ZipTip C18 micro columns were from Millipore, Bedford, MA, USA.

### Animals

Male Wistar rats (250–300 g) (Charles River, Lecco, Italy) were kept one per cage in a temperature-controlled room at 28°C under a 12-h light, 12-h dark cycle. Three groups of rats were used throughout: namely, standard chow diet-fed control rats (referred to as N), high-fat diet-fed rats (referred to as HFD), and long-term (4 weeks) T2-treated HFD rats (referred to as HFD+T2). Each group consisted of six animals. T2 was administrated i.p. at the dose of 25 μg/100 g body weight (Lanni et al., 2005; de Lange et al., 2011; Moreno et al., 2011). During the 4 weeks of treatment, N and HFD rats were sham-injected.

In agreement with previous observations (Lanni et al., 2005; Silvestri et al., 2010; de Lange et al., 2011; Moreno et al., 2011), at the end of the treatment period, the HFD rats were significantly overweight and had higher plasma levels of triglycerides, cholesterol, and alanine aminotransferase (ALT), when compared to the N controls (Table 1). On the other hand, the HFD+T2 rats reached a body weight not significantly different from that of the N animals and had normalized plasma levels of triglycerides, cholesterol, and ALT (Table 1). As already reported (Lanni et al., 2005; Moreno et al., 2011), T2 treatment did not significantly alter heart and lean body mass (i.e., skeletal muscle weight/body weight) (Table 1). Plasma cholesterol, triglycerides and ALT levels were determined by standard procedures.

**Table 1:** Body weight and serum parameters in N, HFD and HFD+T2 rats.

	***N***	**HFD**	**HFD + T2**
Body weight (g)	410 ± 10a	456 ± 15b	421 ± 11a
Heart weight (g)	1.15 ± 0.09a	1.23 ± 0.10a	1.19 ± 0.08a
Gastrocnemius muscle weight (g)	2.30 ± 0.08a	2.55 ± 0.10b	2.34 ± 0.06a
GW/BW (mg/g)	5.54 ± 0.059a	5.59 ± 0.035a	5.56 ± 0.003a
Cholesterol (mg/dl)	59 ± 5a	75 ± 6b	61 ± 3a
Triglycerides (mg/dl)	115 ± 10a	195 ± 25b	135 ± 25a
ALT (U/L)	41 ± 2a	51 ± 4b	42 ± 1a

*Results are expressed as means ± SD; n = 6. Numbers labeled with dissimilar letters are significantly different (P < 0.05). GW/BW, gastrocnemius muscle weight (mg)/body weight (g)*.

At the end of the treatment, rats were anesthetized, and then killed by decapitation. Tissues were excised, weighed, immediately frozen in liquid nitrogen, and then stored at −80°C for later processing. We focused our attention on gastrocnemius muscle, a mixed-type muscle containing regions of slow- and fast-twitch fibers.

All animal treatments and experimental protocols were approved by the Committee on the Ethics of Animal Experiments of the University of Napoli Federico II (Italy) and the Italian Minister of Health (approval number 41469/2011), and are in strict accordance with European directives (86/609/CEE).

### Mitochondrial preparation

Gastrocnemius muscle mitochondria were isolated at 8,000 × g by differential centrifugation (Lombardi et al., 2009). Briefly, tissue fragments were gently homogenized in 10 vol of an isolation medium consisting of 220 mM mannitol, 70 mM sucrose, 20 mM Tris–HCl, 1 mM EDTA, and 5 mM EGTA (pH 7.4) supplemented with 0.5% w/v BSA. The homogenate was centrifuged at 500 × g for 10 min, at 4°C, allowing the isolation of nuclei and cell debris. The resulting supernantant was centrifuged at 8,000 × g. The crude mitochondrial pellet was then washed twice, suspended in a minimal volume of isolation medium, and either kept on ice for respiratory parameter measurements or immediately frozen at −80°C for later processing.

Mitochondrial and total tissue protein content were determined by the DC method (Bio-Rad) in mitochondrial preparation and homogenate, respectively. The protein recovery of the mitochondria-enriched fraction was on average of about 1 mg/g tissue, without significant changes among the groups.

### Protein extraction and two-dimensional gel electrophoresis (2D-E)

2D-E of mitochondrial soluble proteins was performed essentially as previously reported (Lombardi et al., 2009). Briefly, crude mitochondrial pellets were homogenized in 0.25 mL of 8.3 M urea, 2 M thiourea, 2% w/v CHAPS, 1% w/v DTT, 2% v/v IPG buffer (pH 3–10). The extracts were shaken vigorously for 30 min, at 4°C, followed by a 30-min centrifugation at 10,000 × g. Protein concentration was evaluated by the DC method (Bio-Rad). Protein extracts were prepared for each animal, and everyone was assessed separately. Protein samples (650 μg) were applied to immobilized pH 3–10 non-linear gradient strips (17 cm) (Bio-Rad). The second-dimensional separation was performed by using 12% T SDS-polyacrylamide gels. Protein spots were stained using colloidal Coomassie blue, according to the manufacturer's instructions. Electronic images of the gels were acquired by means of a calibrated GS-800 densitometer (Bio-Rad), and analyzed by using PDQuest software (Bio-Rad) (Supplementary Data 1). Spots for which the *P*-value was <0.05 and with at least a 2-fold variation in pairwise comparisons of corresponding volumes between selected experimental groups were considered to display a significant abundance difference.

### Protein digestion, mass spectrometry analysis, and protein identification

Spots from 2D-E were manually excised from gels, triturated, and washed with water. Proteins were *in-gel* reduced, S-alkylated, and digested with trypsin, as previously reported (Scippa et al., 2010). Protein digests were subjected to a desalting/concentration step on μZipTipC18 pipette tips (Millipore) before nano-liquid chromatography (nLC)-electrospray ionization (ESI)-linear ion trap (LIT)-tandem (MS/MS) mass spectrometry analysis. nLC-ESI-LIT-MS/MS analysis was performed on a LTQ XL mass spectrometer (Thermo Fischer Scientific, USA) equipped with a Proxeon nanospray source connected to an Easy-nanoLC (Proxeon, Odense, Denmark). For chromatographic separation, an Easy C18 column (100 × 0.075 mm, 3 μm) (Thermo Fischer) was used at a flow rate of 300 nL/min with the following solvents: A, 0.1% formic acid in water; B, 0.1% formic acid in acetonitrile. Eluent gradient consisted in the following steps: 5–35% B over 10 min, 35–95% B over 2 min, 95% B for 12 min. Raw nLC-ESI-LIT-MS/MS data were searched for protein identification through MASCOT software v2.4.06 (Matrix Science, UK) (Cottrell, 2011), using a *Rattus norvegicus* non-redundant sequence database (NCBI 2012). The following parameters were set for database searching: a mass tolerance value of 2 Da for-precursor ion and 0.8 Da for MS/MS fragments, trypsin as proteolytic enzyme, a missed-cleavages maximum value of 2, Cys carbamidomethylation and Met oxidation as fixed and variable modifications, respectively. Protein candidates with more than 3 significant peptides (*P* < 0.05) with an individual MASCOT score > 30 were further evaluated by comparison with their calculated mass and pI-values, using the experimental values obtained from 2D-E. Protein identity was definitively assigned if the emPAI^1st^ to emPAI^2nd^ ratio observed was >1.5 (Shinoda et al., 2010).

**Figure 1:**
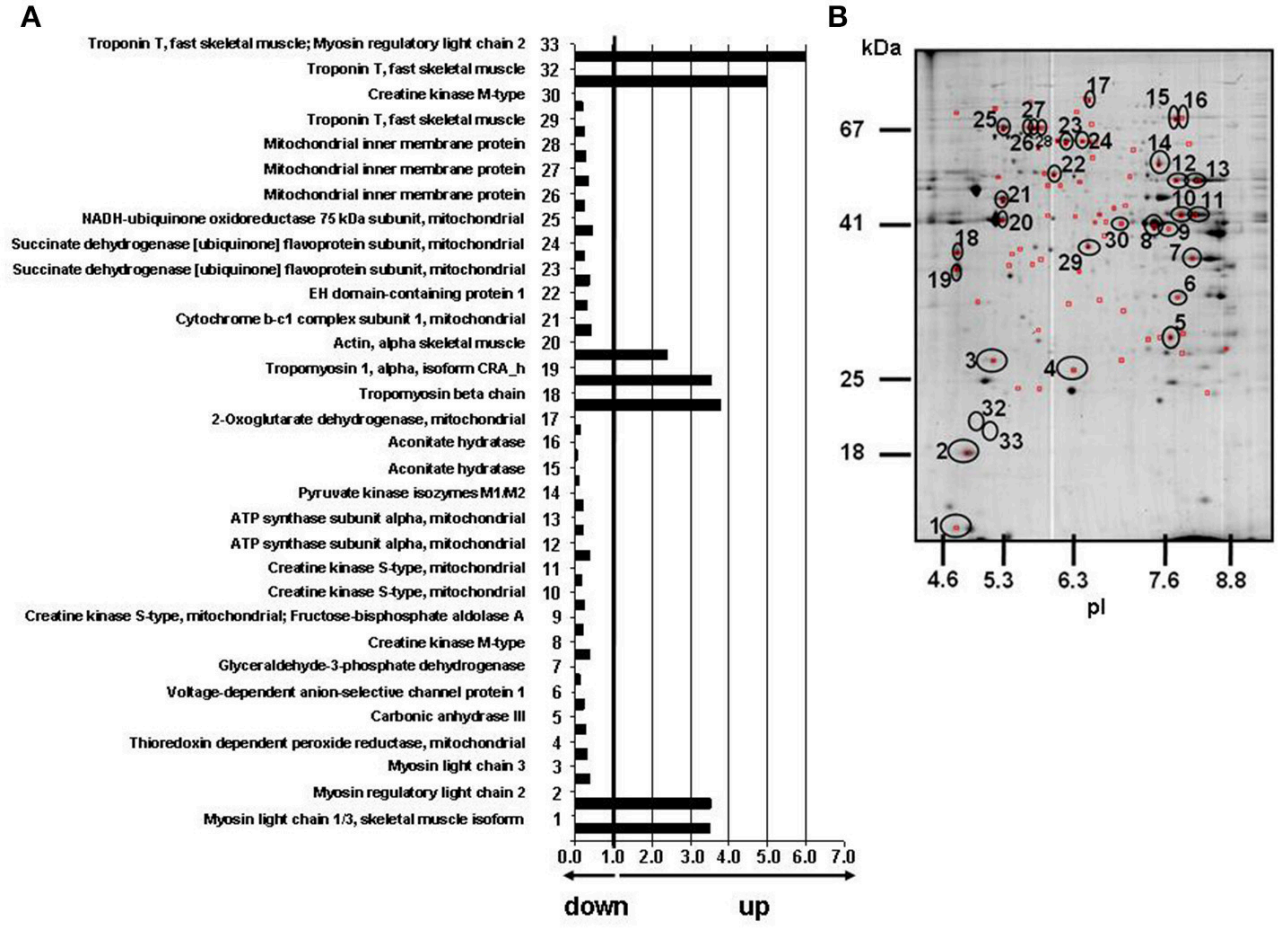
Effects of T2-treatment on gastrocnemius muscle mitochondrial proteome. **(A)** Histogram of protein abundance ratio HFD+T2 vs. HFD. **(B)** Gastrocnemius muscle mitochondria protein profiling by 2D-E. The 32 circled and numbered spots showing differential representation after T2-treatment (vs. HFD) [at least a 2-fold variation in pairwise comparison of corresponding volumes and a statistical significance of at least 95% (*P* < 0.05)], were identified by mass spectrometry analysis. 2D-E was performed using a non-linear pH range of 3–10 in the first dimension (17 cm strips) and SDS-PAGE (12% T) in the second. Protein loading was 650 μg, and the gels were stained using colloidal Coomassie blue.

MS-based protein identification revealed the presence of 2D-E spots that were associated with structural proteins (Figure 1 and Supplementary Data 1–3). The latter accounted for about 4% of the number of total spots. Densitometric analysis estimated their overall contribution to the total gel density, which was close to 4% in both N and HFD, and about 12% in HFD+T2. Thus, the yield of mitochondrial proteins obtained for HFD+T2 was about 8% lower than that of N and HFD.

### *In silico* biological analysis

Differentially represented proteins from 2D-E were input into the IPA platform (Ingenuity® Systems Ltd., USA) for the identification of functions and canonical pathways differing between the treatments. The cut-off values used were 1.5 and 0.05 for the fold change and *P*-values, respectively. In addition, the Ingenuity Pathways Knowledge Base (IPKB) was used to analyze the whole list of differentially represented proteins in the three conditions, in terms of molecular interrelations (networks) based on their connectivity. To build networks, the program utilizes the IPKB containing large numbers of individually modeled relationships between genes (obtained from the literature). The algorithm then determines a statistical score for each network. This is done by comparing the number of focus genes that contribute to a given network relative to the total number of occurrences of those genes in all networks or pathways stored in the IPKB. Then a score is assigned to each network. The score is the negative log of *P*, and it denotes the likelihood that the focus genes in the network might be found together by chance. Therefore, scores of 2 have an at least 99% confidence of not being generated by chance alone. In addition, the biological functions assigned to each network are ranked according to the significance of that biological function to the network. A Fisher's exact test is used to calculate p, indicating the probability that the assignment of the biological function to that network might be explained by chance alone.

### Preparation of gastrocnemius total lysates and nuclei lysates for western blotting

For Western blotting analysis, gastrocnemius muscles or crude nuclear pellets were homogenized in lysis buffer containing 20 mM Tris-HCl, pH 7.5, 150 mM NaCl, 1 mM EDTA, 1 mM EGTA, 2.5 mM Na_2_H_2_P_2_O_7_, 1 mM b-CH_3_H_7_O_6_PNa_2_, 1 mM Na_3_VO_4_, 1 mM PMSF, 1 mg/ml leupeptin, and 1% (w/v) Triton X-100 by using an Optima TLX Ultraturrax (Beckman Coulter, Milan, Italy), then centrifuged at 13,400 × *g* for 10 min, at 4°C. Protein concentration was determined by using the DC method (Bio-Rad).

List of used antibodies: anti-α-Tubulin antibody - Loading Control (ab4074) abcam (rabbit polyclonal); anti-β-actin antibody (ab8227) abcam (rabbit polyclonal); anti-DNM1L antibody (ab56788), abcam (mouse monoclonal); anti-Mitofusin 2 antibody (ab56889), abcam (mouse monoclonal); anti-PGC-1 α antibody (AB3242), Merck (rabbit polyclonal); anti-I-kappa-B-alpha p-IKb antibody (ab12135) abcam (mouse monoclonal); anti-Nuclear factor NF-kappa-B p65 subunit antibody (sc-8008) Santa Cruz Biotecnology (mouse monoclonal).

To verify equal loading between the lanes, samples were loaded in duplicate, with one gel stained with Coomassie blue. Proteins were detected by a chemiluminescence protein-detection method based on the protocol supplied with a commercially available kit (Millipore) and by using the indicated secondary antibodies. Proteins in total tissue extracts were normalized based on β-actin content. Proteins in nuclear extracts were normalized on α-tubulin content. Signals were quantified by means of a ChemiDoc^TM^ XRS densitometer (Bio-Rad), using a dedicated software (QuantityOne, Bio-Rad).

### Blue native (BN)-page and histochemical staining

Solubilization of mitochondrial membranes by detergents, BN-PAGE, staining, and densitometric quantification of oxidative phosphorylation complexes were performed essentially as described by others (Schägger, 1995), with minor modifications. Briefly, the mitochondria-containing sediment was suspended in a low-salt buffer (50 mM NaCl, 50 mM imidazole, pH 7.0), and added of 10% w/v dodecyl-maltoside (final concentration) for solubilization of individual respiratory chain complexes). Immediately after the electrophoretic run, enzymatic colorimetric reactions were performed essentially as reported by others (Zerbetto et al., 1997). After gel scanning, the areas of the colored bands were expressed as absolute values (arbitrary units).

### Mitochondrial H_2_O_2_ release and detection of carbonylated proteins

Mitochondrial H_2_O_2_ production was measured by the Amplex Red-horseradish peroxidase method (Barja, 1998). Horseradish peroxidase (2 U/ml) catalyzes the H_2_O_2_-dependent oxidation of non-fluorescent Amplex Red (80 μM) to fluorescent resorufin red (Zhou et al., 1997). Fluorescence was followed at an excitation wavelength of 540 ± 20 nm and an emission wavelength of 590 nm ± 20 using 96-well black plates and a fluorescence microplate reader (Tecan Infinite 200, Switzerland). The slope of the increase in fluorescence was converted to the rate of H_2_O_2_ production according to a standard curve. All of the assays were performed in respiratory buffer (80 mM KCl, 50 mM Hepes, 1 mM EGTA, 5 mM KH_2_PO_4_, 2 mM MgCl_2_, pH 7) supplemented with 0.3% BSA, at 37°C.

Carbonylated proteins in total tissue were measured using the OxyBlot protein oxidation detection kit (Chemicon, Billerica, MA, USA). The detection kit utilizes an immunoblot that quantifies the level of carbonylated proteins following their reaction with 2,4-dinitrophenylhydrazine to generate corresponding 2,4-dinitrophenylhydrazone (DNPH) derivatives. The resulting dinitrophenyl-derivatized protein samples were blotted onto a membrane filter, incubated with a peroxidase-antibody conjugate that binds to the dinitrophenyl moiety of the protein and goat anti-rabbit IgG, and visualized with chemiluminescent reagents. Protein expression was quantified by densitometry and normalized based on β-actin.

### Ultrastructural analysis of mitochondria

Transmission electron microscopy was performed on sections of gastrocnemius muscles (*n* = 3) for morphological and morphometric mitochondrial analysis. For each animal, a small fragment of gastrocnemius muscle was dissected and immediately fixed in 2% glutaraldehyde, 2% formaldehyde in 0.1 M PB, pH 7.4, overnight, at 4°C. Muscle samples were then cut into smaller fragments measuring about 1 mm^3^ and postfixed in 1% OsO_4_ for 60 min, at 4°C; then, they were dehydrated in acetone and embedded in an Epon-Araldite mixture. To verify longitudinal orientation and section quality, 1 μm-thick sections were cut and stained with 1% toluidine. Thin sections were obtained with an ultramicrotome (2088 Ultratome V, LKB Bromma, Sweden), stained with lead citrate and examined with a Philips CM10 transmission electron microscope (Philips, Eindhoven, The Netherlands).

For each animal, 7–10 mitochondria-rich muscle fibers were analyzed. To study subsarcolemmal (SS) mitochondria, regions containing numerous SS mitochondria were selected and photographs at 7900x for morphometric analysis (500 SS mitochondria per animal were measured). Two subpopulations of intermyofibrillar (IMF) mitochondria were equally examined and photographed, i.e., “peripheral” IMF mitochondria (located between myofibrils at 6–8 sarcomeres away from the plasma membrane) and “central” IMF mitochondrial (located between myofibrils of sarcomeres in the central part of the fiber). For both subpopulations of IMF mitochondria, 1,000 mitochondria per animal were measured.

Mitochondrial size measurements were obtained using analySIS® image analysis system (Soft Imaging System GmbH, Münster, Germany) by manually tracing outlines of SS and IMF mitochondria on 7900x micrographs. Surface area was reported in squared nanometers (nm^2^); Feret's diameter represents the longest distance (nm) between any two points within a given mitochondrion (Picard et al., 2013). Computed values were imported into Microsoft Excel and Prism 5 (GraphPad Software) for data analysis. Statistical significance was evaluated based on 95% confidence interval (CI) of the mean. To produce frequency distributions of each morphological parameter, each mitochondrion was assigned to one of 30 bins of equal size and proportions were determined (relative frequency distribution), yielding frequency distribution per experimental condition (as mean of the relative frequency distribution of three animals).

### Light microscopy

Gastrocnemius muscle samples obtained from rats (*n* = 5) in the same experimental conditions were fixed by immersion in 4% formaldehyde in 0.1 M PB, pH 7.4. After washing in this buffer overnight, the samples were dehydrated in a graded series of ethanol solutions and embedded in paraffin blocks ready for light microscopy. Adjacent serial sections were cut for immunohistochemistry and to assess morphology (by hematoxylin/eosin staining).

### Fiber-type specific immunolocalization of adipocyte differentiation-related protein (ADRP)

To study the fiber-type specific ADRP localization, 3 adjacent serial sections (4 μm-thick) were used to localize: (i) ADRP with a polyclonal anti-ADRP antibody (GP40, mN1, diluted 1:5,000), raised in the guinea pig (Fitzgerald Industries Int, Concord, MA); (ii) myosin heavy chain type I (MHC Ib) with a monoclonal anti-slow myosin antibody (clone NOQ7.5.4D, diluted 1:6,000) (GeneTex Irvine, CA, USA) and, (iii) myosin heavy chain type II (MHC IIb) with a monoclonal anti-fast myosin antibody (Clone MY-32, diluted 1:4,000) (Sigma–Aldrich). Bound antibody was finally stained by the avidin-biotin-peroxidase complex (ABC) method (Hsu et al., 1981) with ABC complex (Vectastain ABC Elite kit, Vector Labs, Burlingame CA) and diaminobenzidine hydrochloride as chromogen (Sigma). Sections were then counterstained with hematoxylin and mounted in Eukitt (Kindler, Freiburg, Germany). Specificity tests were performed by omitting the primary antiserum in the staining and by using preimmune serum instead of the primary antiserum.

### Statistical analysis

Reported values are the means ± SD. All the data, except for those obtained in the 2D-E analysis (in which the used image software applies by default Student's *t*-test for pairwise comparison) were evaluated by ANOVA followed by the Newman–Keuls test, with the minimum level of significance being *P* < 0.05.

## Results

### Mitochondrial soluble proteome in the gastrocnemius muscle of N, HFD, and HFD+T2 rats

To gain insights into the effects exerted by HFD and T2-treatment on mitochondrial phenotype, solubilized proteins from gastrocnemius muscle mitochondria of N, HFD, and HFD+T2 rats were subjected to a 2D-E-based proteomic analysis. At the detection-limits set, the software counted 230 common matched proteins among the various electrophoretic maps. Pairwise comparisons were performed to analyze the differential expression pattern associated with HDF and T2-treatment. Limiting our interest to a differential expression of at least 2-fold and a statistical significance of at least 95% (*P* < 0.05), 9 (about 4% of total entries) and 78 spots (about 34% of total entries) resulted to show significant quantitative changes in HFD vs. N and in HFD+T2 vs. HFD, respectively. Numerical difference in terms of spots meeting the criteria of differential expression in the comparison HFD vs. N and HFD+T2 vs. HFD likely reflected the diverse nature of the two treatments under study as well as their diverse impact on the gastrocnemius muscle mitochondrial proteome.

All spots differentially represented in HFD+T2 vs. HFD mitochondria were digested with trypsin, subjected to nano-LC-ESI-LIT-MS/MS analysis and further assayed for their nature by bioinformatics of resulting data (Figure 1 and Supplementary Data 2, 3). Only, 32 spots were identified, with the remaining ones not providing significant results based on their low quantity or the occurrence of similar amounts of comigrating components in the same gel portion. The theoretical molecular mass (Mr) and isoelectric point (pI) values of each identified component corresponded roughly to its position on the 2D-E gel. When proteins were identified as multiple spots on the same map, putatively reflecting the occurrence of post-translational modifications, the pattern of changes was fairly similar among the various species.

**Figure 2:**
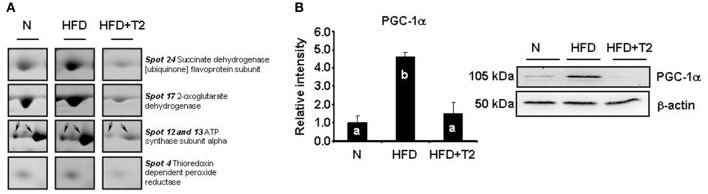
Examples of differential representation of mitochondrial proteins and PGC-1α in gastrocnemius muscle of N, HFD, and HFD+T2 rats. **(A)** Representative subsections of 2D-E images are shown as examples of differential representation of mitochondrial proteins between experimental groups. Spot numbering refers to Figure 1. **(B)** Representative Western blot analysis of the expression levels of PGC-1α in gastrocnemius muscle of N, HFD, and HFD+T2 rats. Data were normalized to the value obtained for N animals, set as 1, and presented separately for each treatment (means ± SD; *n* = 4/6). Bars labeled with dissimilar letters are significantly different (*P* < 0.05).

On the basis of 2D-E and mass spectrometry data, it was evident that T2-treatment significantly altered the mitochondrial protein representation profile displayed by the gastrocnemius muscle under the HFD condition. Mitochondria from HFD+T2 rats, when compared to those from HFD ones, were characterized by a general down-representation of proteins and enzymes involved in intra-mitochondrial oxidative catabolism (Figure 1). In line with a tissue displaying a shift toward a more glycolytic phenotype (Moreno et al., 2011), representation levels of carbonic anhydrase III (spot 5), creatine kinase S-type mitochondrial (spots 9, 10, and 11), creatine kinase M-type (spots 8 and 30), ATP synthase subunit alpha (spots 12 and 13, Figure 2A), aconitate hydratase (spots 15 and 16), 2-oxoglutarate dehydrogenase (spot 17, Figure 2A), cytochrome b-c1 complex subunit 1 (spot 21), succinate dehydrogenase [ubiquinone] flavoprotein subunit (spots 23 and 24, Figure 2A) and NADH-ubiquinone oxidoreductase 75 kDa subunit (spot 25) were significantly decreased following T2-treatment (Figure 1). The levels of the thioredoxin-dependent peroxide reductase (also known as peroxideroxin 3) (spot 4, Figure 2A) were also decreased by T2-treatment. This important mitochondrial anti-oxidant enzyme is involved in the redox regulation and protection of radical-sensitive enzymes from the oxidative damage induced by radical-generating systems as well as in the activation of NF-kB in the cytosol. In parallel, the gastrocnemius muscle content of PGC-1α, was significantly increased by HFD feeding and normalized by T2-treatment (Figure 2B).

### *In silico* analysis of the mitochondrial proteins differentially expressed in HFD+T2 rats

**Figure 3:**
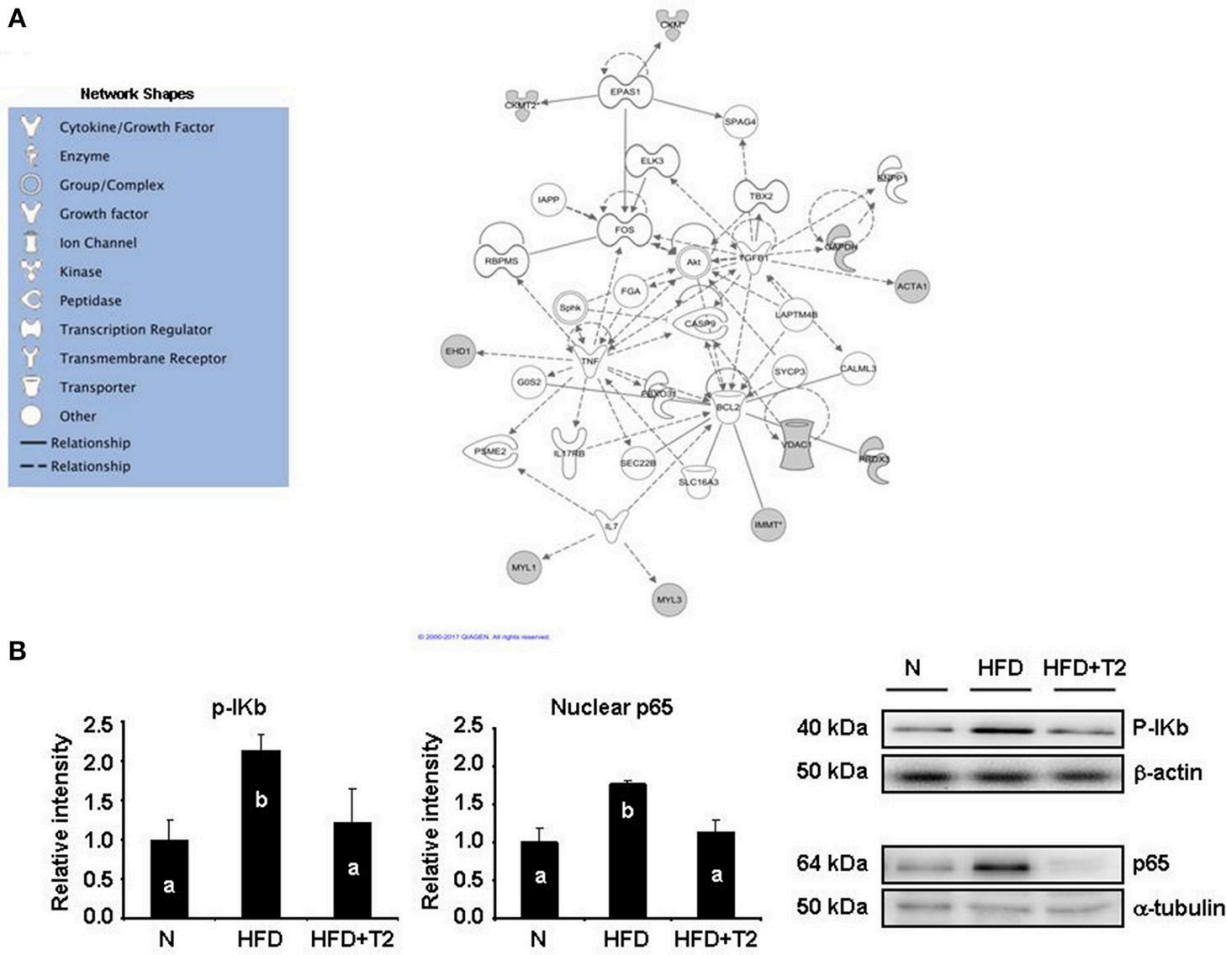
*In silico* protein network analysis. **(A)** Network representation of the molecular relationships between identified, differentially represented gastrocnemius muscle proteins (Ingenuity Systems Ltd.). Gene products are represented as nodes, and the biological relationship between two nodes is represented as an edge (line). Indirect interactions without physical contact appear as broken lines, whereas direct interactions requiring direct physical contact between nodes appear as solid lines. All edges shown are supported by at least 1 reference from the literature, from a textbook, or from canonical information stored in the Ingenuity Pathways Knowledge Base. Human, mouse, and rat orthologs of a gene are stored as separate objects in IPKB, but are represented as a single node in the network. For clarity, network shapes are shown. **(B)** Western blot analysis of the expression levels of p-Ikb and p65 subunit of NF-kB in gastrocnemius muscle of N, HFD, and HFD+T2 rats. p65 expression was measured in nuclear extracts. Gastrocnemius muscle proteins were normalized based on β-actin. Nuclear proteins were normalized based on α-tubulin. Data were normalized to the value obtained for *N* animals, set as 1, and presented separately for each treatment (means ± SD; *n* = 4/6). Bars labeled with dissimilar letters are significantly different (*P* < 0.05).

To define the molecular events possibly activated in the gastrocnemius muscle following T2-treatment, we next analyzed proteomic results by using the IPA platform to identify canonical-pathways and functions eventually affected. Most significant expression changes involved canonical pathways associated with mitochondrial dysfunction, citrate cycle (TCA cycle), oxidative phosphorylation, and glycolysis/gluconeogenesis (Supplementary Data 4A). As far as it concerns canonical functions, affected and interesting ones were energy production, carbohydrate metabolism, as well as cell signaling (Supplementary Data 4B). In addition, the IPKB was used to analyze the list of differentially expressed proteins also in terms of molecular interrelations (networks) according to the previous literature. Figure 3A illustrates the highest-scoring network (score, 21) obtained for the identified proteins. Four central nodes were observable, namely tumor necrosis factor (TNF), B-cell lymphoma 2 (BCL2), transforming growth factor beta 1 (TGFB1), and FBJ osteosarcoma oncogene (FOS).

These nodes were interconnected with several other identified proteins, suggesting a simultaneous effect of T2-treatment on substrate metabolism- as well as inflammation–related pathways. In particular, FOS is directly interconnected with Endothelial PAS domain-containing protein 1 (EPAS1) which, in turn, appears directly interconnected with creatine kinase S-type (CKMT2) and creatine kinase M-type (CKM). These interconnections suggest T2-treatment-induced effects on energy transduction in skeletal muscle and modulation of oxygen-regulated genes. TGFB1 appears correlated with glyceraldehyde-3-phosphate dehydrogenase (GAPDH) and actin (ACTA1), two proteins that may be considered markers of metabolic and structural changes induced by T2 in the gastrocnemius muscle. Moreover, recent studies have demonstrated that TGFB1, a part from controlling proliferation and differentiation, may interfere with mitochondrial oxidative phosphorylation and generation of reactive oxygen species (Abe et al., 2013).

The voltage-dependent anion-selective channel protein 1 (VDAC1), the thioredoxin dependent peroxide reductase (PRDX3) and the mitochondrial inner membrane protein (IMMT) converge on BCL2. This sub-network, based on the known roles played by these proteins in cell functions, suggests T2-treatment associated effects on cell redox homeostasis, mitochondrial cristae morphology, and mitochondrial membranes permeability.

The above discussed three nodes converge on the fourth, TNF. This last is interconnected with the EH domain-containing protein 1 and, through the BCL2 node, with interleukin 7 (IL7). As stated above, this region of the obtained network strongly suggests an interference of the T2-treatment with inflammation–related pathways, specifically with NF-kB, a central factor controlling inflammatory cytokines, antioxidants, stress proteins, and survival factors. We thus measured Ikb phosphorylation (p-Ikb), index of the activation of the pro-inflammatory factor NF-kB, and the nuclear levels of the corresponding p65 subunit. As reported in Figure 3B, HFD markedly increased (vs. N) both Ikb phosphorylation and the nuclear levels of p65 subunit. T2-treatment, on the other hand, normalized both parameters (Figure 3B).

### Individual respiratory complex activity in the gastrocnemius muscle of N, HFD, and HFD+T2 rats

**Figure 4:**
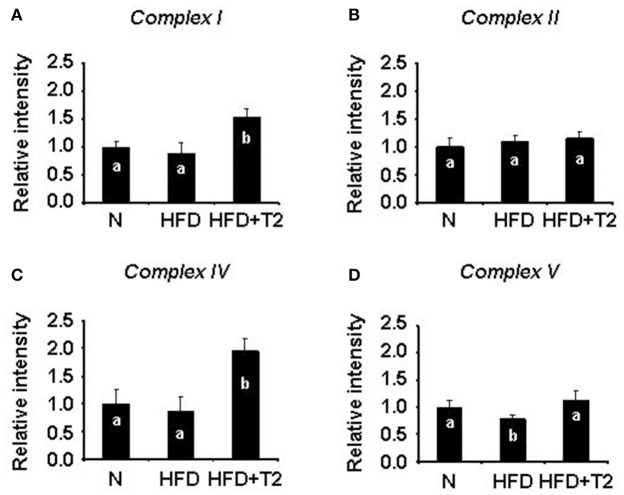
BN-PAGE-based *in-gel* activity of individual mitochondrial respiratory complexes from gastrocnemius muscle of N, HFD, and HFD+T2 rats. **(A)** *In-gel* activity of complex I. **(B)** *In-gel* activity of complex II. **(C)** *In-gel* activity of complex IV. **(D)** *In-gel* activity of complex V. Protein extracts were prepared for each animal, and each individual was assessed separately. Data were normalized to the value obtained for N animals, set as 1, and presented separately for each treatment (means ± SD; *n* = 6). Bars labeled with dissimilar letters are significantly different (*P* < 0.05).

We next investigated on the effect of HFD and T2 in terms of respiratory chain complex activities. Gastrocnemius muscle mitochondrial respiratory chain complexes of N, HFD, and HFD+T2 rats resolved by BN-PAGE were analyzed for their individual *in-gel* activities. HFD regimen for 4 weeks, while not changing the activity of complexes I, II, and IV (Figures 4A–C) resulted in a reduction of the activity of complexes V (vs. N and HFD+T2) (Figure 4D). T2-treatment was effective at stimulating complex I and IV (vs. N and HFD) (Figures 4A,C) and in normalizing complex V activity (Figure 4D). No significant differences between the experimental groups were observed as far as it concerns complex II activity (Figure 4B).

### Mitochondrial H_2_O_2_ release and protein carbonylation in the gastrocnemius muscle of N, HFD, and HFD+T2 rats

**Figure 5:**
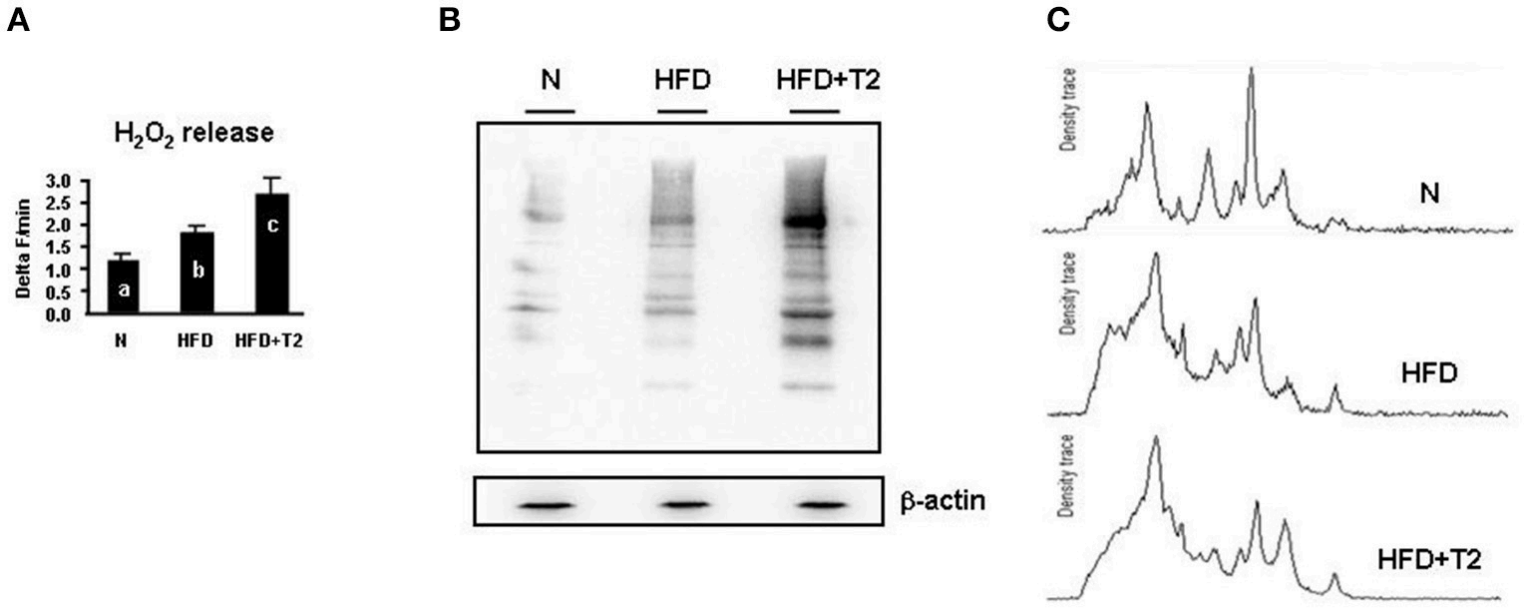
Effects of HFD and T2-treatment on mitochondrial H_2_O_2_ release and protein carbonylation. **(A)** Mitochondrial H_2_O_2_ release (Delta F/min) in gastrocnemius muscle mitochondria of N, HFD and HFD+T2 rats. Bars labeled with dissimilar letters are significantly different (*P* < 0.05). **(B)** Representative immunoblot showing protein carbonylation in gastrocnemius muscle of N, HFD, and HFD+T2 rats. **(C)** Representative density traces for carbonylated protein bands are also reported.

To study the functional consequences of the so far described proteomic and respiratory features of gastrocnemius muscle mitochondria, mitochondrial H_2_O_2_ release (an indirect index of mitochondrial superoxide production *in vitro*), and tissue levels of carbonylated proteins were determined in N, HFD and HFD+T2 gastrocnemius muscle samples. When compared to N controls, gastrocnemius muscle of HFD animals showed a significantly higher mitochondrial H_2_O_2_ release (+59% vs. N). This parameter resulted to be further increased in HFD+T2 animals (+ 51% vs. HFD; +141% vs. N) (*P* < 0.05) (Figure 5A). Accordingly, when compared to N controls, gastrocnemius muscle of HFD animals showed significantly increased levels of carbonylated proteins (Figures 5B,C). In agreement with H_2_O_2_ concentration values, the levels of carbonylated proteins were further increased in HFD+T2 animals (Figures 5B,C).

### Mitochondrial morphology and dynamics in gastrocnemius muscle of N, HFD, and HFD+T2 rats

**Figure 6:**
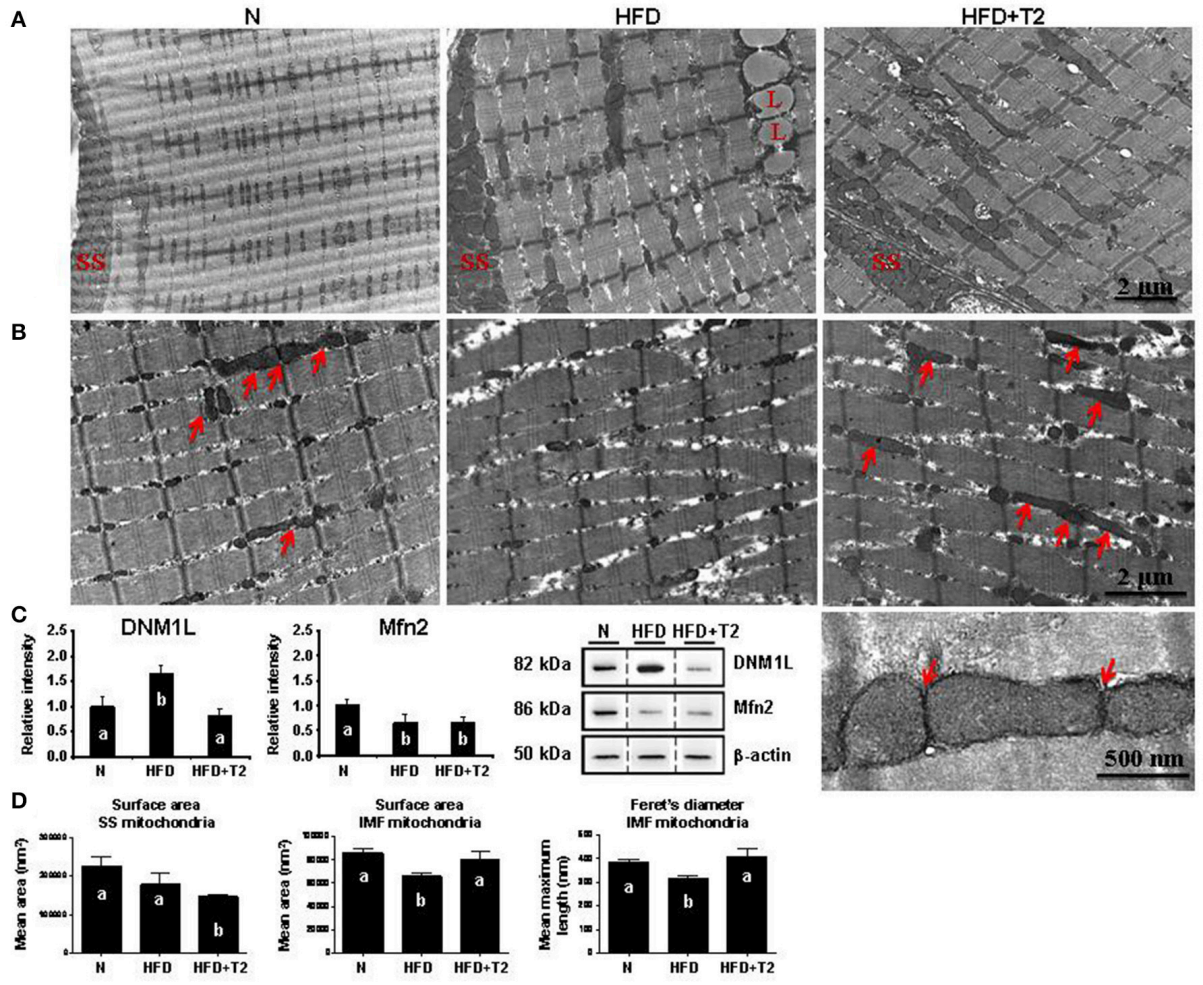
Effects of HFD and T2-treatment on mitochondrial morphology and dynamic markers in gastrocnemius muscle of N, HFD, and HFD+T2 rats. **(A)** Peripheral and **(B)** central intermyofibrillar (IMF) regions of gastrocnemius muscle fibers in N, HFD, HFD+T2 rats. Longitudinal views in electron microscopy revealed the arrangement of subsarcolemmal (SS) and IMF mitochondria. Many continuous, elongated mitochondria were observed in N and HFD+T2 conditions (arrows). Lipid vacuoles are visible in muscle fibers of HFD animals (L). Higher magnification (bottom right image) demonstrated tubular and continuous mitochondria spanning the Z-lines as well as and electron-dense contact sites at the outer membranes of adjacent mitochondria (arrows) in gastrocemius muscle fibers of HFD+T2 rats. **(C)** Western blot analysis of DNM-1L and Mfn 2 protein levels in gastrocnemius muscles of N, HFD, and HFD+T2 rats. Data were normalized to the value obtained for *N* animals, set as 1, and presented separately for each treatment (means ± SD; *n* = 4/6). Bars labeled with dissimilar letters are significantly different (*P* < 0.05). The vertical dotted lines indicate cuts in the membranes. **(D)** Mean surface area and Feret's diameter for SS and IMF mitochondria in gastrocnemius muscle of N, HFD, and HFD+T2 rats (*n* = 3). Bars labeled with dissimilar letters are significantly different (*P* < 0.05).

We next examined whether mitochondrial morphology could be affected in response to metabolic overload in HFD rats, and questioned whether the T2- specific induced changes in gastrocnemius muscle fiber composition (Moreno et al., 2011) and mitochondrial features could also be associated with differences in the representation level of central players in mitochondrial fusion/fission machinery. The electron microscopy-driven ultrastructural analysis of gastrocnemius muscles showed that HFD feeding altered both the sarcomere arrangement and the morphology of intermyofibrillar (IMF) mitochondria (HFD vs. N) (Figures 6A,B). Specifically, myofibrils appeared narrowed and splitted in HFD rats, with areas of focal loss of myofilaments, while IMF mitochondria looked like swelled with dissolved/degranulated membranes. No continuous, elongated and interacting mitochondria across the Z-line were observed; in particular, the classical/normal pairwise arrangement of IMF mitochondria across the Z-line was profoundly altered (Figure 6B).

On the other hand, T2-treatment reduced the abnormal structural changes produced by HFD in sarcomere arrangement and preserved the mitochondrial morphology (Figures 6A,B). Indeed, HFD+T2 gastrocnemius muscle samples contained many elongated and tubular IMF mitochondria layered between sarcomeres and spanning the Z-lines in the central region of fibers. In particular, electron-dense structures, physically linking the outer membranes of these tubular mitochondria, were often observed (Figure 6B, see the high magnification), thus suggesting that the IMF mitochondria of HFD+T2 gastrocnemius muscles were strictly interacting one another, likely forming inter-mitochondrial junctions (IMJs) (Picard et al., 2015).

The observation in longitudinal views of the skeletal muscle fibers revealed that, as far as it concerns the subsarcolemmal (SS) mitochondria, independently of the experimental group, they were clustered beneath the plasma membrane, but appeared heterogeneous in size both in HFD and in HFD+T2. These morphological details were supported by Western blotting analysis on total gastrocnemius lysates that revealed that HFD feeding, while significantly increasing the expression levels of DNM-1L, simultaneously decreased those of Mfn2 (vs. N) (Figure 6C), likely pointing toward a HFD-induced imbalance of the mitochondrial dynamic machinery in favor of fission. T2-treatment resulted in a normalization of the expression levels of DNM-1L and a slight but not significant increase of those of Mfn2 (vs. HFD) (Figure 6C), thus suggesting that T2 might mitigate the HFD-induced perturbation of the mitochondrial dynamic machinery likely preventing fission.

Mitochondrial morphometric analysis in both SS and IMF regions of gastrocnemius muscle fibers revealed differential effects of HFD and T2-treatment (Figure 6D). HFD produced (vs. N) no significant difference in the mean mitochondrial area in SS regions (Figure 6D). Specifically, SS mitochondria in HFD appeared disorganized and heterogeneous in size, with some large mitochondria flanked by numerous small ones (for additional images and frequency distribution of the surface area of the SS mitochondria in the experimental groups, see Supplementary Data 5).

Instead, HFD significantly reduced the value of both the surface area and the Feret's diameter of IMF mitochondria (Figure 6D). Considering that IMF mitochondria represent the most abundant mitochondrial population in the skeletal muscle fibers, these data appear to be strictly in line with the increased expression levels of DNM-1L observed in HFD rats. On the other hand, T2-treatment lead to: (i) SS mitochondria with a significantly reduced surface area vs. both N and HFD and, (ii) a normalization of both the surface area and the Feret's diameter of IMF mitochondria (Figure 6D and Supplementary Data 5). In the SS region of gastrocnemius muscles of HFD+T2 rats, large mitochondria were observed but these appeared rare, smaller than those observed as large in N and HFD, and flanked by numerous small ones (Supplementary Data 5). Once again, the morphometric data appeared coherent with those of protein expression corroborating the idea that T2 might prevent, at least in the IMF region, the HFD-induced structural mitochondrial aberrations.

Further, we performed a separate morphometric analysis distinguishing between “peripheral” and “central” IMF mitochondria (for the definition of “peripheral” and “central” IMF mitochondria, see the Material and Methods section). HFD significantly reduced (vs. N) the mitochondrial size (i.e., both the surface area and the Feret's diameter) in the “central” but not in the “peripheral” IMF mitochondria (Supplementary Data 6). Analogously, T2-treatment, while tended to preserve a tubular morphology in all the IMF mitochondrial subpopulations, significantly normalized the mean Feret's diameter only in the “central” IMF mitochondria (Supplementary Data 6).

### Subcellular distribution of ADRP immunoreactivity around lipid droplets in gastrocnemius muscles of N, HFD, and HFD+T2 rats

**Figure 7:**
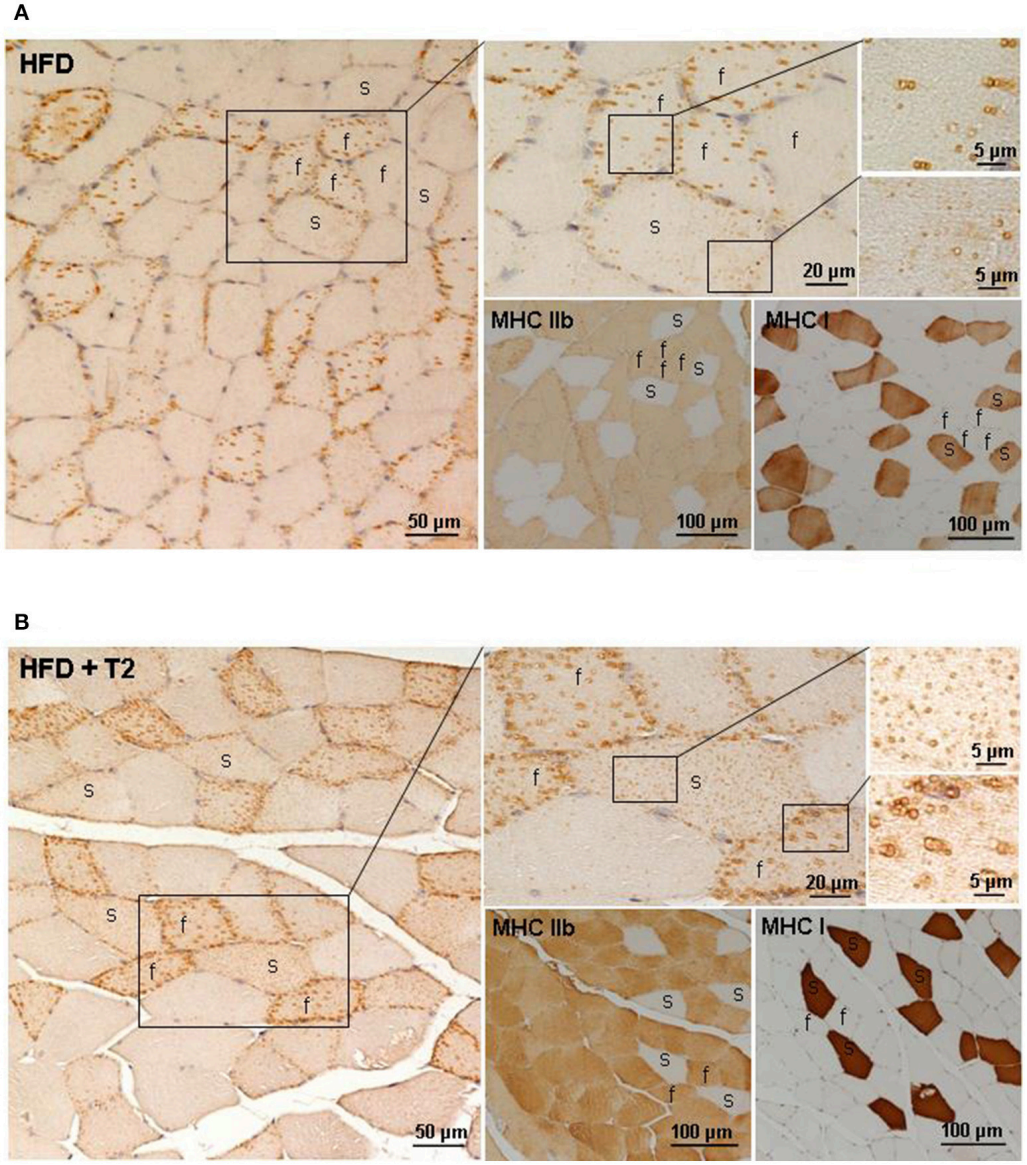
Fiber type-specific ADRP immunoreactivity in gastrocnemmius muscle of HFD and HFD+T2 rats. ADRP immunohistochemistry in gastrocnemius muscle of HFD **(A)** and HFD+T2 **(B)** rats. Fiber type-specific ADRP-immunoreactive lipid droplets were obtained using adjacent serial sections (4 μm-thick) to stain ADRP, MHC Ib, and MHC IIb proteins. Distribution of ADRP protein around LDs in a fast (f) and slow (s) fiber was shown by a higher magnification of the same framed area. In HFD rats, ADRP immunolabeling coated some but not all LDs, both in fast and slow fibers; in HFD+T2 rats, all small LDs (in slow fibers) and large (in fast fibers) lipid vacuoles showed an intense ADRP-staining.

Several evidences support metabolic interactions between mitochondria and lipid droplets (LDs) to balance IMCL accumulation, hydrolysis, oxidation as well as lipotoxicity. ADRP is one of the most abundantly expressed LD-coating proteins in the skeletal muscle, where it has been suggested to control proper lipid storage. In this study, we examined the effects of HFD and T2 on the fiber type-specific ADRP immunostaining of the intermyofibrillar lipid droplets. In the gastrocnemius muscle of HFD rats, the ADRP immunoreactivity appeared predominantly around some but not all the large lipid droplets stored in fast/glycolytic fibers; the slow/oxidative fibers being very weakly ADRP-immunoreactive (Figure 7A). After T2-treatment, all the lipid droplets were intensely ADRP immunostained both in fast/glycolytic and slow/oxidative fibers (Figure 7B), thus indicating differential and opposing effects of HFD and T2-treatment on the ADRP-dependent control of lipid storage.

## Discussion

In mammalian skeletal muscle, while it has well-established that high-fat feeding impairs insulin-stimulated glucose transport and uptake rates (Hansen et al., 1998; Tremblay et al., 2001; Krisan et al., 2004; Yaspelkis et al., 2007), the causative role of mitochondria dysfunction in this impairment remains not clearly elucidated. In the present study, we report that HDF-induced IMLC accumulation, slow oxidative fiber enrichment, and insulin sensitivity reduction (i.e., reduced insulin-stimulated AKT activation) (Moreno et al., 2011) were actually associated with important alterations in the network of factors controlling mitochondrial morphogenesis machinery and metabolic functions, when compared with standard diet conditions. The HFD regimen while noninvasively affecting the gastrocnemius muscle mitochondrial proteome, in terms of number of spots differentially represented in HFD vs. N, reduced individual complex V *in-gel* activity. In parallel, HFD also induced an increase in the representation levels of PGC-1α, one of the key factor modulating skeletal muscle fiber-type switching (Lin et al., 2002), mitochondrial biogenesis, and adaptive thermogenesis, and of enzymes involved in oxidative metabolism in skeletal muscle (Lin et al., 2002; Puigserver and Spiegelman, 2003). HFD mitochondria (vs. N ones) also showed an increased H_2_O_2_ release that, together with augmented levels of carbonylated proteins in tissues, suggests a mitochondrial function impairment and a likely HFD-induced intramitochondrial damage, which is a well-known marker of IR in skeletal muscle (Kelley et al., 2002; Petersen et al., 2004). Indeed, when compared to N, IMF mitochondria from HFD rats had reduced surface area and Feret's index appearing smaller and fragmented, strictly paralleling the fission state (i.e., higher expression levels of DNM-1L and the lower ones of Mfn2). These data were in line with *in vitro* and *in vivo* studies showing fatty acid-induced mitochondrial fragmentation and increased mitochondrion-associated DNM-1L levels, oxidative stress, mitochondrial depolarization, loss of ATP production, and reduced insulin-stimulated glucose uptake (Jheng et al., 2012; Holmström et al., 2013). The HFD-induced increase in IkB activation and the concomitant increase of the nuclear content of the subunit p65 of NF-κB supported the involvement of inflammatory pathways in the impairment of skeletal muscle sensitivity to insulin (Coletta and Mandarino, 2011).

On the other hand, T2-treatment prevented the HFD-induced-effects on mitochondrial biogenesis and dynamics, as well as on inflammation. As revealed by the proteomic analysis, muscle mitochondria from HFD+T2 rats displayed: (i) reduced protein representation levels of enzymes involved in oxidative catabolism and redox regulation (e.g., thioredoxin dependent peroxide reductase); (ii) increased levels of tissue protein carbonylation, index of oxidative stress and damage. Moreover, muscle mitochondria from HFD+T2 rats showed some specific features significantly differing from those observed both in N and in HFD, that is stimulated *in-gel* activities of individual respiratory complex I and IV, and increased H_2_O_2_ release.

In fast fibers, it has been suggested that greater ROS production per mitochondrial unit may be required to maintain proper redox-dependent signaling, despite low mitochondrial content (Anderson and Neufer, 2006; Picard et al., 2008), and may contribute, together with other factors, to trigger adaptive mitochondrial biogenesis, specifically in glycolytic muscles (Picard et al., 2012). Importantly, it has to be considered that the mitochondrial H_2_O_2_ release may depend on a plethora of mechanisms (Kwong and Sohal, 1998; Kadenbach, 2003; Balaban et al., 2005), among which worth mentioning is the activity of antioxidant enzymes that is significantly lower in glycolytic fibers compared with oxidative ones (Picard et al., 2012). Accordingly, proteomic analysis revealed here reduced levels of the mitochondrial thioredoxin dependent peroxide reductase in the gastrocnemius muscle from HFD+T2 rats vs. the HFD ones. This could be in part a consequence also of the existing differences between HFD and HFD+T2 in the expression level of PGC-1α that regulates, not only mitochondrial biogenesis, but also the mRNA expression level of many ROS detoxifying enzymes (St-Pierre et al., 2006). Thus, the H_2_O_2_ buffering capacity per mitochondrial unit (likely related to antioxidant enzyme expression and activity) may differ considerably in HFD+T2 muscle compared with HFD one, and may account for the differences observed in H_2_O_2_ emitting potential between HFD+T2 and HFD muscles. Accordingly, the higher levels of protein carbonylation and mitochondrial H_2_O_2_ release in gastrocnemius of T2-treated animals vs. HFD may be interpreted as the result of a scaled/reduced antioxidant defenses. In this context, the observed T2-associated increase in ROS production should be considered important to maintain normal functions (i.e., glucose utilization and insulin sensitivity) of glycolytic fibers under a HFD regimen, putatively eliciting a mitohormetic action (Ristow and Zarse, 2010; Ost et al., 2015). In fact, transiently increased levels of oxidative stress have been suggested to reflect potentially health-promoting processes at least in regards to prevention of IR and T2D (Ristow et al., 2009). Indeed, it has been reported that exogenous antioxidant supplementation in humans blunts the benefits of exercise training in terms of insulin sensitivity and response of mitochondrial biogenesis signaling (Ristow et al., 2009; Strobel et al., 2011). Of note, the higher levels of mitochondrial H_2_O_2_ release in gastrocnemius of T2-treated animals (vs. both N and HFD ones) appears to be strictly correlated with both the higher percentage of fast glycolytic fibers and the higher levels of the sarcolemma GLUT4 protein levels (Moreno et al., 2011). In addition, despite the T2-treatment dependent increase in tissue protein carbonylation, a normalized expression profile of pro-inflammatory markers (i.e., nuclear levels of NF-kB p65 subunit) was detected, thus suggesting a lack of tissue damage, at least in terms of inflammation. It has been reported that the administration of T2 to streptozotocin-induced diabetic rats, eliciting a marked SIRT1 activation-dependent renoprotective action, strongly attenuated the diabetic nephropathy-associated increase in NF-kB p65 subunit acetylation/activation (Shang et al., 2013). These and our data, indicate that T2 might interfere with the NF-kB signaling pathway eliciting anti-inflammatory actions.

As far as it concerns the mitochondrial morphology, mitochondria from HFD+T2 rats were significantly different from those of HFD ones in the IMF regions of the fibers; they appeared as tubular, elongated and tethered organelles above all across the Z-line of the sarcomeres and fused. Mitochondrial dynamics can be linked to the balance between energy demand and nutrient supply; thus, both HFD- and T2-dependent changes in mitochondrial architecture might represent mechanisms for bioenergetic adaptation to changed metabolic demands. Since a causative link between mitochondrial dynamics and IR has been established, also demonstrating that the inhibition of mitochondrial fission protects muscle cells against mitochondrial dysfunction and IR *in vitro*, and improves muscle insulin signaling and insulin sensitivity *in vivo* (Jheng et al., 2012), the T2-associated prevention of the increase of the DNM-1L representation levels can be interpreted as a preventing action against mitochondrial fission in favor of insulin signaling in skeletal muscle, despite the HFD regimen.

Skeletal muscle-insulin sensitivity requires a fine balance between lipid storage and oxidation (Aon et al., 2014). ADRP [also known as perilipin 2 (PLIN2)], one of the major LD-coating proteins in skeletal muscle, where it is supposed to control lipid accumulation stabilizing LDs and inhibiting lipolysis (Bosma et al., 2012), has been suggested to be higher in content in circumstances of improved glucose tolerance (Phillips et al., 2005). We report here that the ADRP immunoreactivity in gastrocnemius muscle of HFD specifically appeared mainly distributed around some but not all the large LDs in fast/glycolytic fibers and only very weakly in slow/oxidative ones. Taking into consideration that the HFD gastrocnemius muscle is enriched in slow/oxidative fibers (Moreno et al., 2011), these data further point toward a positive correlation between the skeletal muscle content of ADRP and the *in vivo* muscle insulin sensitivity. On the other hand, the effects on gastrocnemius muscle metabolism of T2 treatment during a HFD regimen included, among the others: (i) a reduction of IMLC accumulation with increased ADRP immunoreactivity around all the IMLC in oxidative and glycolityc fibers; (ii) an enhanced insulin-independent expression and translocation of glucose transporters to the plasma membrane; (iii) an enrichment of glycolytic myofibers; (iv) an amelioration of insulin sensitivity (Moreno et al., 2011). Once again, these results were suggestive of a positive correlation between the skeletal muscle content of ADRP and the *in vivo* insulin sensitivity.

Overall, we have shown here that the T2-induced shift of gastrocnemius muscle toward glycolytic myofibers during a HFD regimen (Moreno et al., 2011) is accompanied by a co-adaptation of mitochondrial structural and functional features. Moreover, T2 likely blunted lipotoxicity and, importantly, did not decrease the skeletal muscle mass, which is a known marker of thyroid hormone–induced sarcopenia. In addition, this work, in line with very recent ones (Zhang and Ye, 2012; Holloszy, 2013; Meng et al., 2013), further supports how the oxidative-to-glycolytic metabolic shift in skeletal muscle is potentially beneficial in hyperglycemic/prediabetic states (de Lange et al., 2011). Nevertheless, concerning the nature of the mechanisms that underlie the described effects, important questions remain to be solved yet. For example, a controversy exists as far as it concerns the possibility that T2 might exert some of its actions in a TR-dependent manner (Mendoza et al., 2013; Navarrete-Ramírez et al., 2014; Orozco et al., 2014; Jonas et al., 2015). Actually, also genomic actions of T2 have been described but, at least in mammals, doses up to 100-fold greater than those of T3 were required to generate comparable effects (Orozco et al., 2014). Thus, a putative interaction of T2 with nuclear TRs cannot be excluded *a priori*, when using treatment protocols with very high doses (Goldberg et al., 2012; Jonas et al., 2015). Indeed, the current literature clearly suggests that the actions of T2 might be dose-, specie-, diet-, and age-specific (Padron et al., 2014; Vatner et al., 2015; Coppola et al., 2016; da Silva Teixeira et al., 2016; for recent review, see Goglia, 2015). At the same time, a direct mitochondrial mode of action of T2 cannot be excluded to explain the results concerning the activity of the respiratory complexes I and IV (Arnold et al., 1998; Davis et al., 2016 and references within). Further studies are being conducting to address these questions.

## Author contributions

ES: designed the experimental approaches, performed proteomic analyses, supervised data elaboration, wrote and revised the manuscript; FC: designed the experimental approaches, performed proteomic analyses, wrote and revised the manuscript; RD: performed electron microscopy-based ultrastructural analysis of mitochondria and fiber-type specific immunolocalization of adipocyte differentiation-related protein, elaborated data, wrote the manuscript; RS: supervised animal care and treatments and revised the manuscript; PdL: designed the experimental approaches, supervised data elaboration and revised the manuscript; MaC: performed proteomic and western blot analyses and revised the manuscript; AS and AMS: performed MS analyses, elaborated data, and revised the manuscript; MC: performed *in silico* analyses, elaborated data, and revised the manuscript; FG and ALa: contributed to the design of the work and revised the manuscript; MM: designed the experimental approaches, supervised data elaboration, wrote and revised the manuscript; ALo: coordinated the experimental procedures and revised the manuscript.

### Conflict of interest statement

The authors declare that the research was conducted in the absence of any commercial or financial relationships that could be construed as a potential conflict of interest.
