# Supplementary material for: 3,5-Diiodo-L-Thyronine Affects Structural and Metabolic Features of Skeletal Muscle Mitochondria in High-Fat-Diet Fed Rats Producing a Co-adaptation to the Glycolytic Fiber Phenotype
Source: Front Physiol. 2018 Mar 9;9:194. doi: 10.3389/fphys.2018.00194 (PMC5854997; doi:10.3389/fphys.2018.00194)

## Slide 1
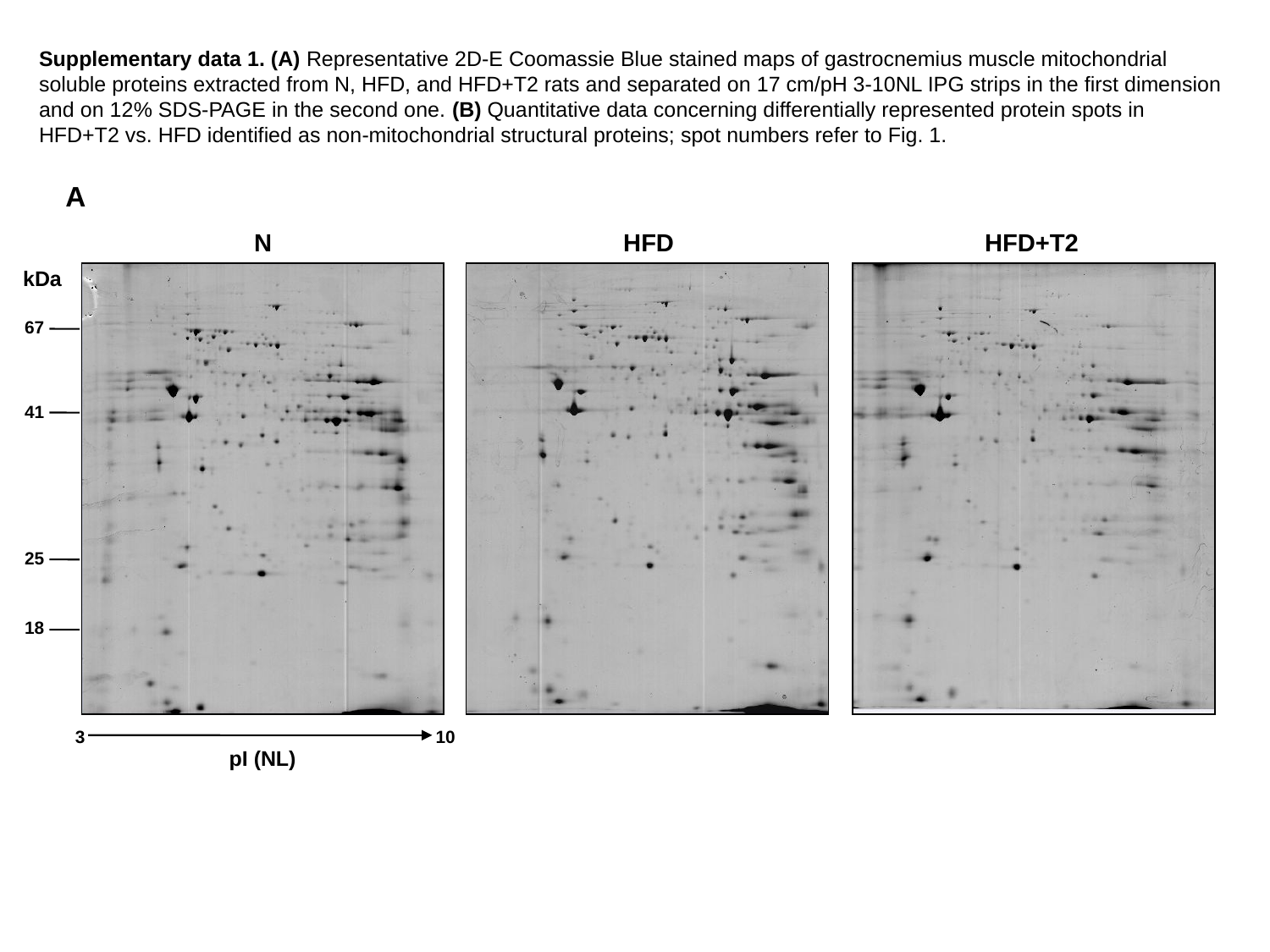

Supplementary data 1. (A) Representative 2D-E Coomassie Blue stained maps of gastrocnemius muscle mitochondrial soluble proteins extracted from N, HFD, and HFD+T2 rats and separated on 17 cm/pH 3-10NL IPG strips in the first dimension and on 12% SDS-PAGE in the second one. (B) Quantitative data concerning differentially represented protein spots in HFD+T2 vs. HFD identified as non-mitochondrial structural proteins; spot numbers refer to Fig. 1.
A
N
HFD
HFD+T2
kDa
67
41
25
18
3
10
pI (NL)

## Slide 2
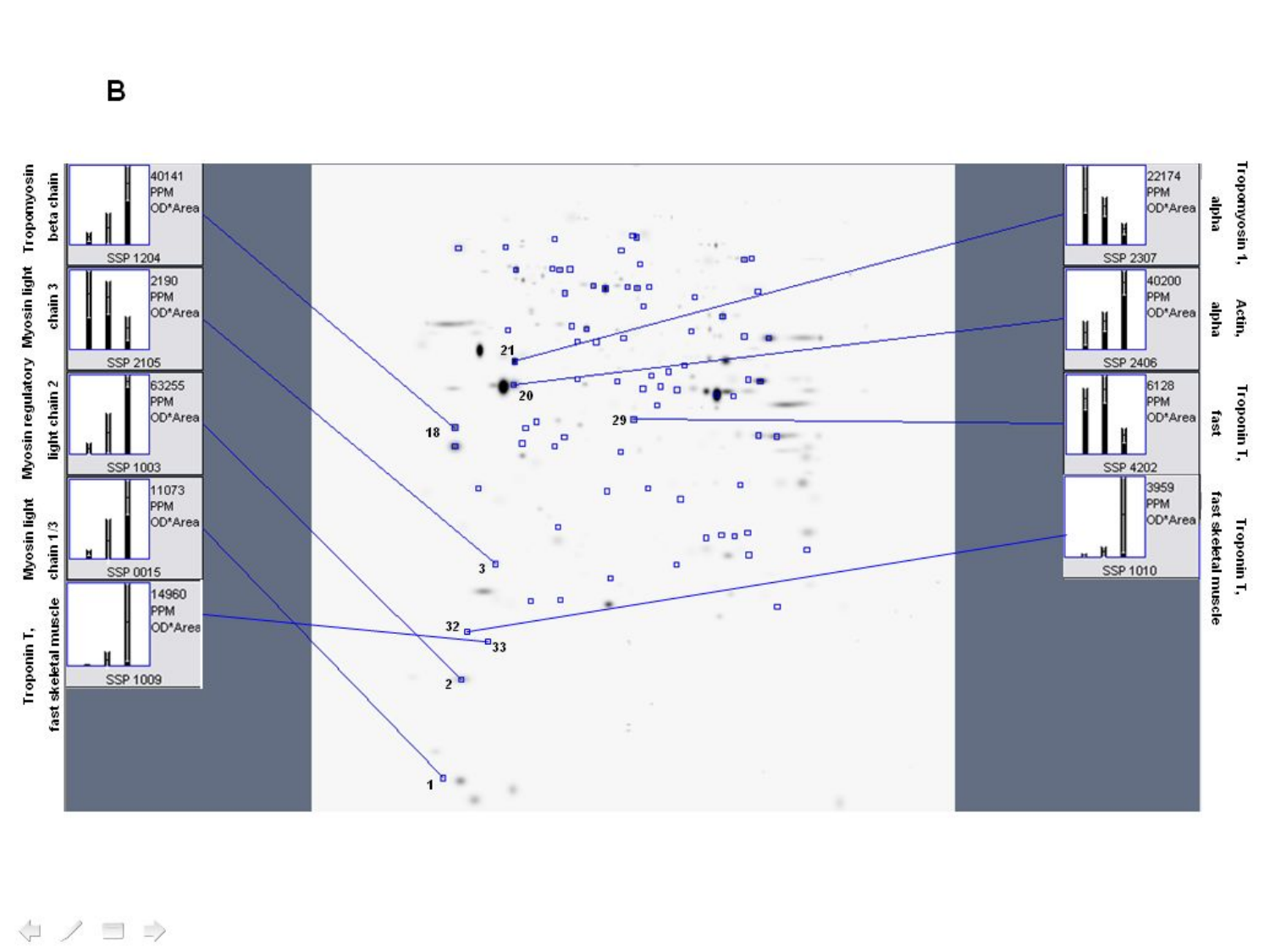

Supplement: Supplementary file 1 [file SupplementaryData1.PPT]
