# Supplementary material for: 3,5-Diiodo-L-Thyronine Affects Structural and Metabolic Features of Skeletal Muscle Mitochondria in High-Fat-Diet Fed Rats Producing a Co-adaptation to the Glycolytic Fiber Phenotype
Source: Front Physiol. 2018 Mar 9;9:194. doi: 10.3389/fphys.2018.00194 (PMC5854997; doi:10.3389/fphys.2018.00194)
Supplement: Supplementary file 4 [file SupplementaryData4.PPT]

## Slide 1
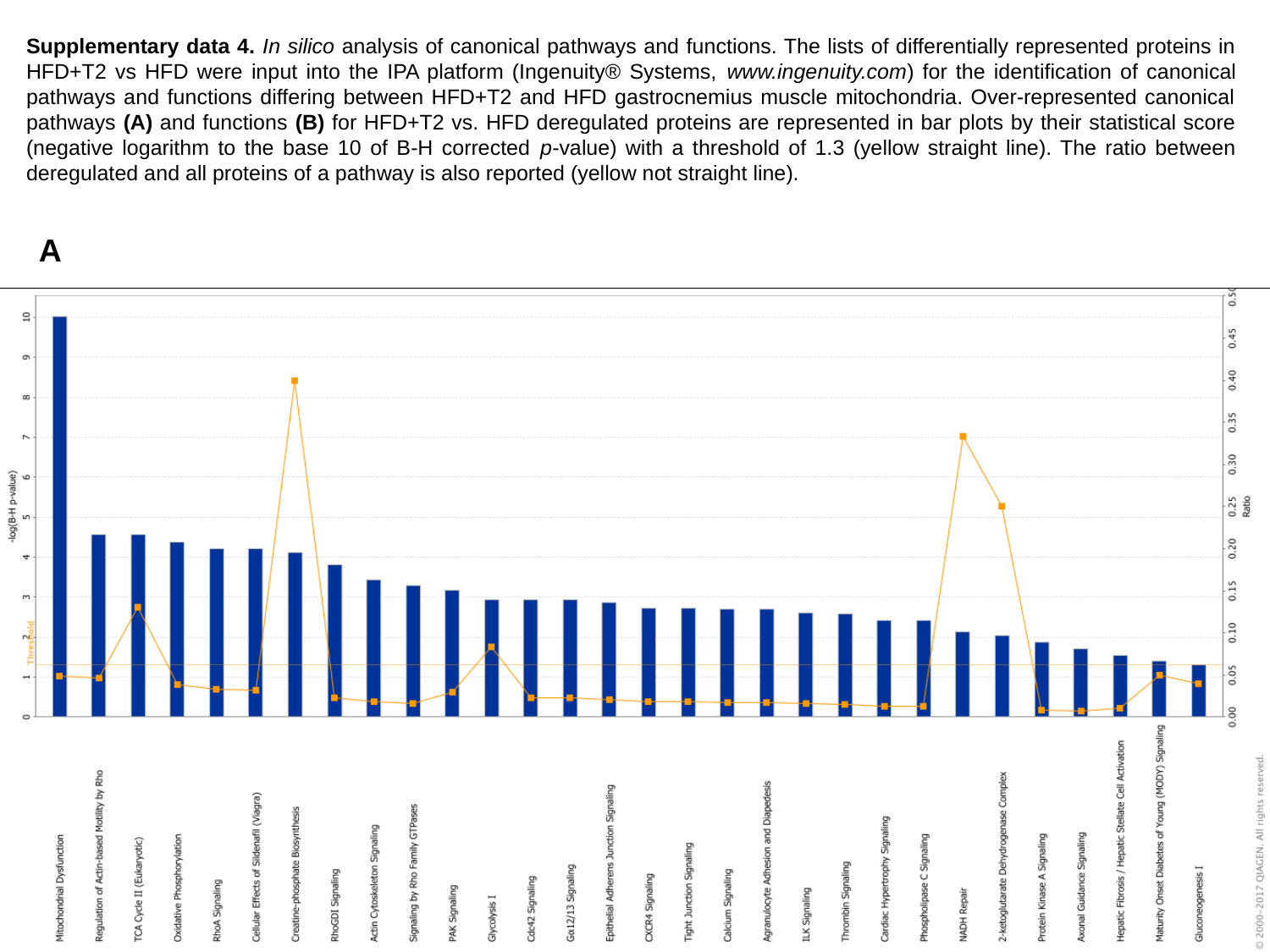

Supplementary data 4. In silico analysis of canonical pathways and functions. The lists of differentially represented proteins in HFD+T2 vs HFD were input into the IPA platform (Ingenuity® Systems, www.ingenuity.com) for the identification of canonical pathways and functions differing between HFD+T2 and HFD gastrocnemius muscle mitochondria. Over-represented canonical pathways (A) and functions (B) for HFD+T2 vs. HFD deregulated proteins are represented in bar plots by their statistical score (negative logarithm to the base 10 of B-H corrected p-value) with a threshold of 1.3 (yellow straight line). The ratio between deregulated and all proteins of a pathway is also reported (yellow not straight line).
A

## Slide 2
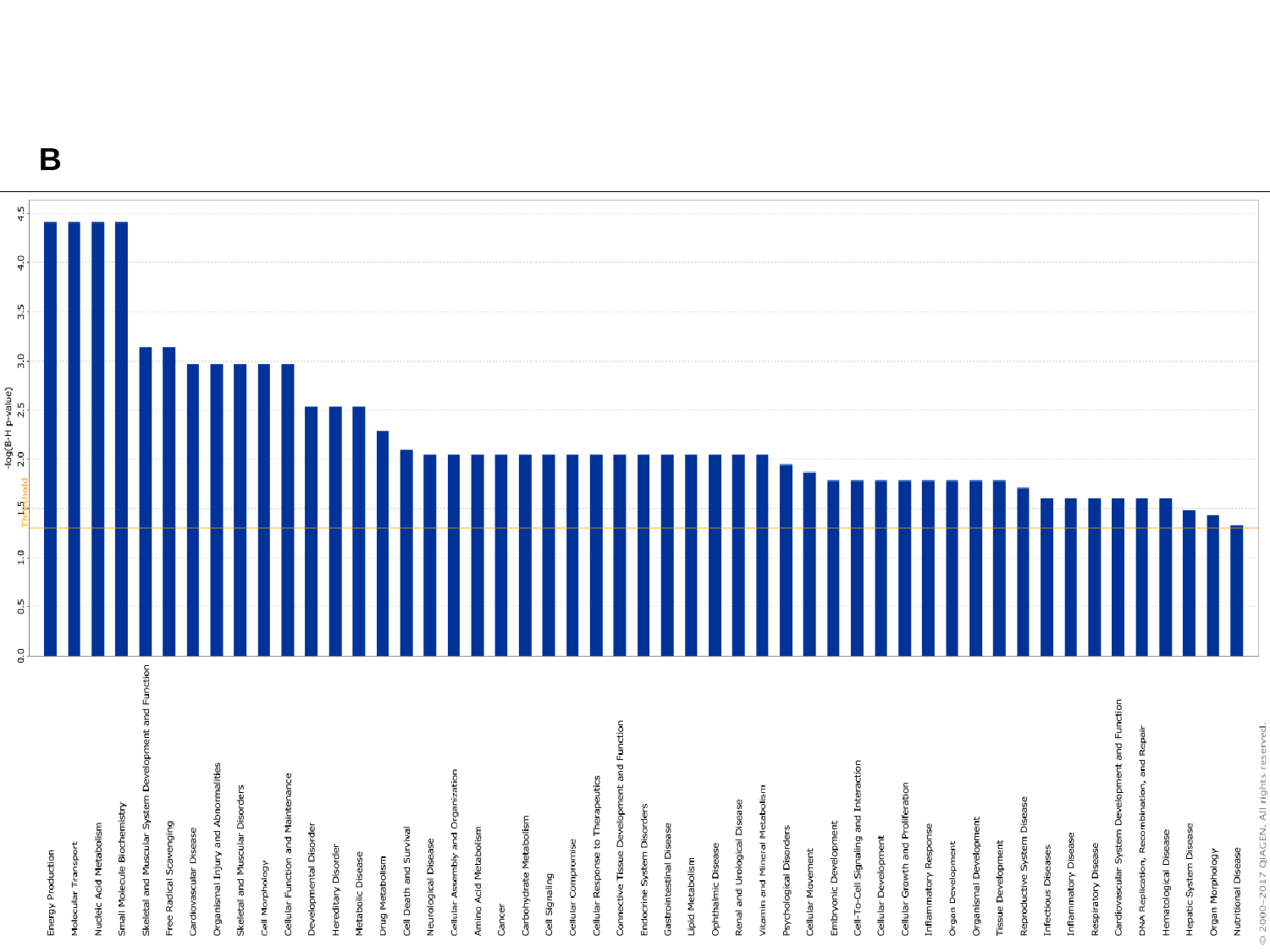

B
