# Supplementary material for: 3,5-Diiodo-L-Thyronine Affects Structural and Metabolic Features of Skeletal Muscle Mitochondria in High-Fat-Diet Fed Rats Producing a Co-adaptation to the Glycolytic Fiber Phenotype
Source: Front Physiol. 2018 Mar 9;9:194. doi: 10.3389/fphys.2018.00194 (PMC5854997; doi:10.3389/fphys.2018.00194)
Supplement: Supplementary file 5 [file SupplementaryData5.PPT]

## Slide 1
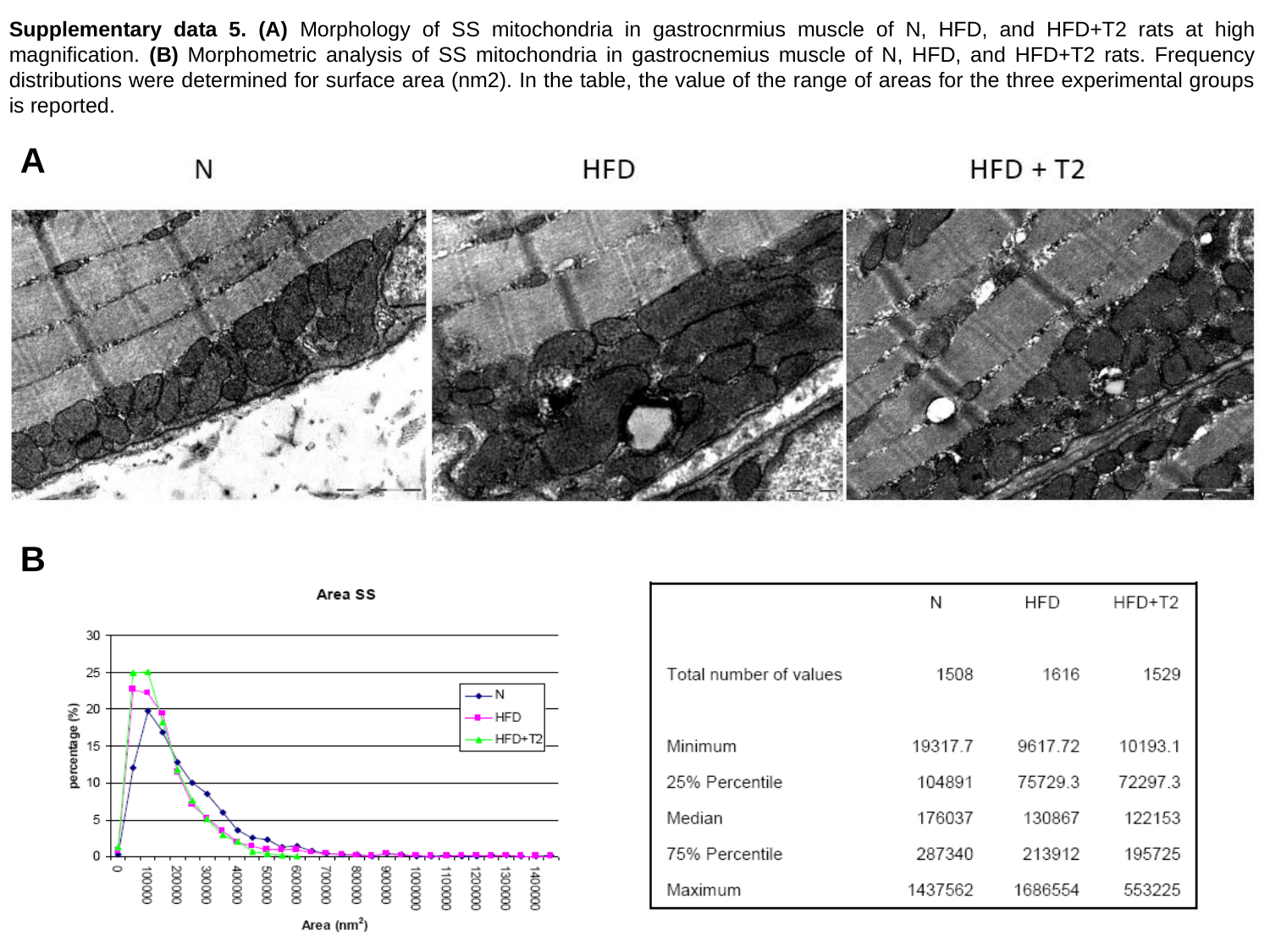

Supplementary data 5. (A) Morphology of SS mitochondria in gastrocnrmius muscle of N, HFD, and HFD+T2 rats at high magnification. (B) Morphometric analysis of SS mitochondria in gastrocnemius muscle of N, HFD, and HFD+T2 rats. Frequency distributions were determined for surface area (nm2). In the table, the value of the range of areas for the three experimental groups is reported.
A
B
