# Supplementary material for: 3,5-Diiodo-L-Thyronine Affects Structural and Metabolic Features of Skeletal Muscle Mitochondria in High-Fat-Diet Fed Rats Producing a Co-adaptation to the Glycolytic Fiber Phenotype
Source: Front Physiol. 2018 Mar 9;9:194. doi: 10.3389/fphys.2018.00194 (PMC5854997; doi:10.3389/fphys.2018.00194)
Supplement: Supplementary file 6 [file SupplementaryData6.PPT]

## Slide 1
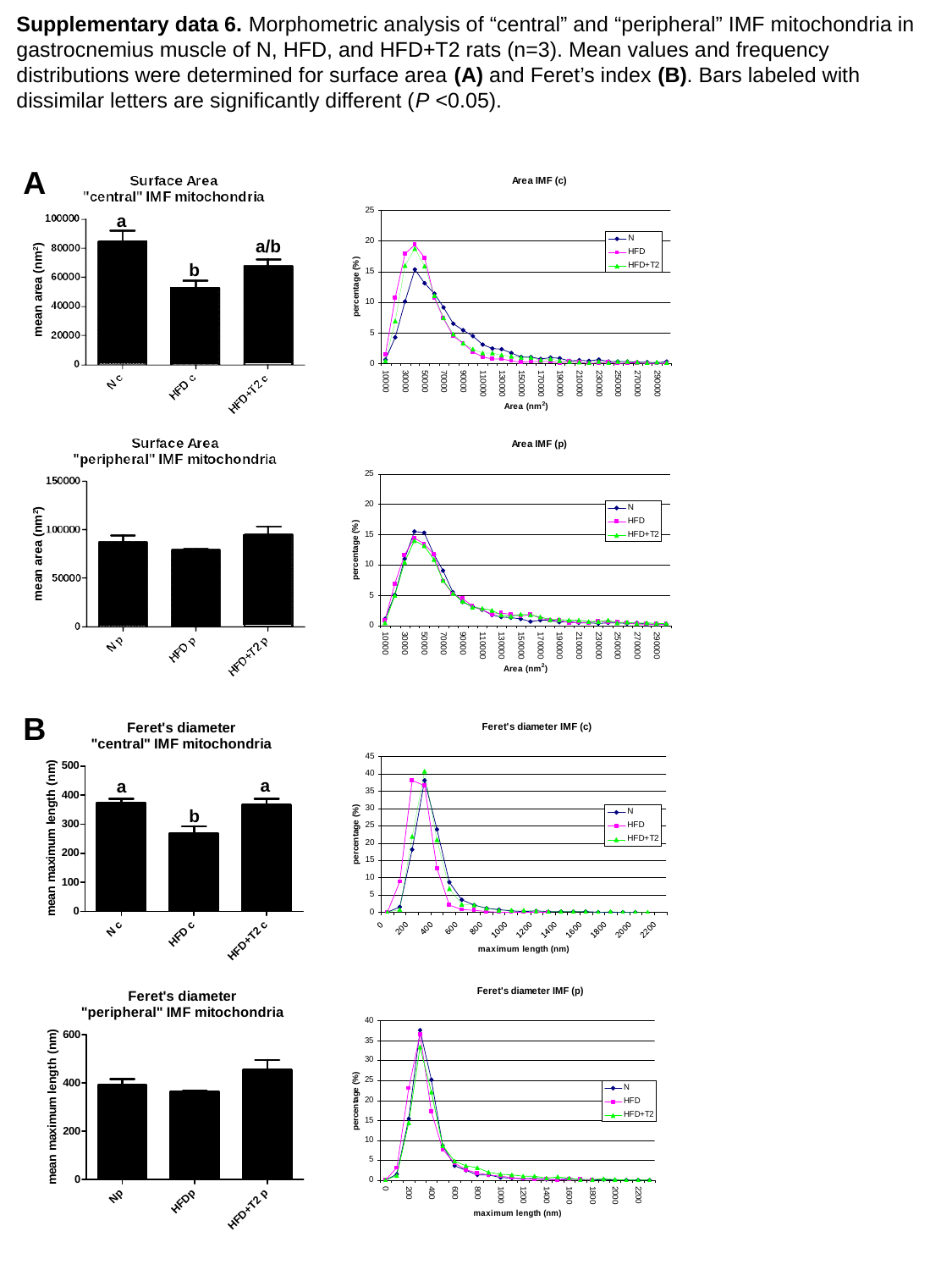

Supplementary data 6. Morphometric analysis of “central” and “peripheral” IMF mitochondria in gastrocnemius muscle of N, HFD, and HFD+T2 rats (n=3). Mean values and frequency distributions were determined for surface area (A) and Feret’s index (B). Bars labeled with dissimilar letters are significantly different (P <0.05).
A
a
a/b
b
mean area (nm2)
mean area (nm2)
B
a
a
b
